# Cell-penetrating peptide conjugates to enhance the antitumor effect of paclitaxel on drug-resistant lung cancer

**DOI:** 10.1080/10717544.2017.1321060

**Published:** 2017-05-04

**Authors:** Ziqing Duan, Cuitian Chen, Jing Qin, Qi Liu, Qi Wang, Xinchun Xu, Jianxin Wang

**Affiliations:** 1 Department of Pharmaceutics, School of Pharmacy, Fudan University & Key Laboratory of Smart Drug Delivery, Ministry of Education, Shanghai, PR China,; 2 Institute of Clinical Pharmacology, Guangzhou University of Traditional Chinese Medicine, Guangzhou, PR China, and; 3 Shanghai Xuhui Central Hospital, Shanghai, PR China

**Keywords:** Paclitaxel, low molecular weight protamine, TAT, conjugate, cell-penetrating peptide, drug-resistant lung cancer

## Abstract

To conquer the drug resistance of tumors and the poor solubility of paclitaxel (PTX), two PTX-cell-penetrating peptide conjugates (PTX-CPPs), PTX-TAT and PTX-LMWP, were synthesized and evaluated for the first time. Compared with free PTX, PTX-CPPs displayed significantly enhanced cellular uptake, elevated cell toxicity, increased cell apoptosis, and decreased mitochondrial membrane potential (Δψm) in both A549 and A549T cells. PTX-LMWP exhibited a stronger inhibitory effect than PTX-TAT in A549T cells. Analysis of cell-cycle distribution showed that PTX-LMWP influenced mitosis in drug-resistant A549T tumor cells via a different mechanism than PTX. PTX-CPPs were more efficient in inhibiting tumor growth in tumor-bearing mice than free PTX, which suggested their better *in vivo* antitumor efficacy. Hence, this study demonstrates that PTX-CPPs, particularly PTX-LMWP, have outstanding potential for inhibiting the growth of tumors and are a promising approach for treating lung cancer, especially drug-resistant lung cancer.

## Introduction

Lung cancer is a fatal disease with high incidence and mortality rates (Siegel et al., 2012). As a cytotoxic microtubule-stabilizing agent extracted from *Taxus brevifolia* (Pacific Yew) (de Hoon et al., 2012) and a commonly used drug to treat lung cancer in the clinic (Xu et al., 2016), paclitaxel (PTX) has made a significant contribution on improving the quality of life and prolonging the survival time of lung cancer patients. However, drug resistance has become a serious challenge in the application of PTX (Zuo et al., 2010; Rasco et al., 2010; Ferrara et al., 2016). The mechanisms of drug resistance can be divided into pump resistance and non-pump resistance (Minko et al., 2013). Pump resistance plays an important role and is primarily caused by membrane-bound active drug efflux pumps such as P-glycoprotein, multidrug resistance-associated proteins, and breast cancer resistance proteins (Patel et al., 2013). As a transmembrane adenosine triphosphate (ATP)-dependent efflux pump presenting in a great diversity of tumors (Yusuf et al., 2003), P-glycoprotein efflux pump is the most widely reported mechanism of PTX resistance (Yusuf et al., 2003; Luqmani, 2005). Overexpression of P-glycoprotein in cancer cells leads to decreased accumulation of PTX and reduced antitumor efficacy. Many studies (Zunino & Capranico, 1990; Nicholson, 2000; Galletti et al., 2007; Vasile et al., 2015) have also revealed that non-pump mechanisms, including but not limited to drug degradation, mutation of tubulin/microtubule system, antiapoptotic defense, and DNA repair, also participate in the development of PTX resistance.

To conquer this problem, many efforts have been made to diminish or circumvent the activity of efflux pumps. The discovery of efflux pump inhibitors has attracted much attention, and co-delivery of anticancer drugs with efflux transporter inhibitors has shown potential for reversing drug resistance in tumors (Patil et al., 2009; Song et al., 2009). However, applications of the inhibitors are hindered by their lack of specificity, poor bioavailability, adverse effects and limited clinical activities (van Zuylen et al., 2000; Pusztai et al., 2005; Kibria et al., 2014). With the development of nanomedicine, some researchers (Susa et al., 2009; Wang et al., 2011) have demonstrated that nanoparticles can help antitumor drugs bypass efflux pumps by preventing the exposure of antitumor drugs and transporting them into cells through endocytic pathway. However, in order to overcome drug resistance using nanocarrier strategies, antitumor drugs should remain incorporated in nanoparticles until internalization (Wang et al., 2015b). Considering most nanoparticles are designed to disassemble at the tumor site and release their cargo, the clinical anti-drug-resistant effect of nanoparticles still needs to be assessed cautiously.

Modifying the structure of anti-cancer drugs is another effective strategy to circumvent drug resistance (Liang & Yang, 2005; Ferrara et al., 2016). According to a recent study (Minko et al., 2013), antitumor drugs excluded by efflux pumps need to conform to three essential properties: having a relatively low molecular weight, capable of mimicing the substrate of an efflux pump and being internalized into cells through passive diffusion. Therefore, modifying the molecular structure of a drug and/or changing the mechanism of drug internalization could, in theory, bypass efflux pumps (Minko et al., 2013; Vargas et al., 2014). Meanwhile, conjugating anti-cancer drugs with functional molecules, such as proteins, peptides, monoclonal antibodies, polymers, glycans, or other molecules, can deliver the drugs into cancer cells through active uptake rather than passive diffusion (Liang et al., 2012; Li et al., 2013; Murakami et al., 2013; Teow et al., 2013; Wang et al., 2015a). Many scientists have focused their attention on developing new antitumor drug conjugates to overcome efflux pumps and thus the drug resistance of tumors. For example, ANG1005, the conjugate of PTX and an angiopep-2 peptide, had the capacity bypassing the P-gp efflux pump and enhancing the ability of PTX to cross the blood–brain barrier (Regina et al., 2008). Han (Han et al*.,* 2014) designed a triphenylphosphonium–doxorubicin conjugate and successfully applied it toward reversing the drug resistance of breast cancer cells. Luo (Luo et al., 2015) synthesized a novel xyloglucan − mitomycin C/doxorubicin conjugate and used it to achieve an extraordinary *in vivo* antitumor effect in multidrug-resistant HepG2 cells.

Cell-penetrating peptides (CPPs) have been used as efficient tools for delivering pharmaceuticals and nanosystems into cells (Xia et al., 2011; Chen et al., 2014; Zhong et al., 2015). Some antitumor drugs, such as doxorubicin (Soudy et al., 2013; Zhang et al., 2013) and methotrexate (Szabo et al., 2016), have been conjugated to CPPs and obtained the ability to overcome drug resistance. As a first-line antitumor drug, PTX has serious limits that need to be addressed. In addition to its limited antitumor effect on drug-resistant cancer cells, the hydrophobicity of PTX is also a problem that needs to be solved when developing PTX formulations. Because most CPPs are hydrophilic, conjugating PTX to CPPs can not only enhance the transport of PTX into tumor cells and then inhibiting drug resistance, but also make PTX-CPPs water-soluble, thus avoiding the use of inorganic solvents, such as Cremophor EL. Among the CPPs, TAT (GGGYGRKKRRQRRR), derived from HIV and discovered as the first CPP, has been widely used to decorate bio-functional molecules and drug delivery systems (Tseng et al., 2002; de la Torre et al., 2014; Song et al., 2015). Low molecular weight protamine (LMWP) (VSRRRRRRGGRRRR) is another newly found CPP and has also been confirmed to have the capacity to enhance the internalization of linked cargos (Xia et al., 2011,2013). Therefore, TAT and LWMP were selected as CPPs to modify PTX in our study.

Hence, two new PTX-CPP conjugates (PTX-TAT and PTX-LMWP) were synthesized for the efficient transduction of PTX into PTX-sensitive (A549) and PTX-resistant lung cancer cells (A549T). The conjugates were evaluated for their intracellular delivery, cell toxicity, induction of cell apoptosis, effect on mitochondrial membrane potential (Δψm) and cell cycle, and *in vivo* antitumor efficacy compared with free PTX in both sensitive and drug-resistant cells.

## Materials and methods

### Materials

LMWP (VSRRRRRRGGRRRR) and TAT (CGGGYGRKKRRQRRR) peptides were purchased from Bankpeptide Biological Technology Co., Ltd. (Hefei, China). Paclitaxel (PTX) was purchased from Dalian Meilun Biotech Co., Ltd. (Dalian, China). *N*-(2-Aminoethyl) maleimide trifluoroacetate salt was purchased from Sigma-Aldrich Co., LLC (St. Louis, MO). Trifluoroacetic acid (TFA), 3-(4,5-dimethylthiazol-2-yl)-2,5-diphenyl tetrazolium bromide (MTT), *N*-hydroxysuccinimide (NHS), *N*-(3-dimethylaminopropyl)-*N*′-ethylcarbodiimide hydrochloride (EDC), and *N,N*-diisopropylethylamine (DIPEA) were purchased from aladdin-e.com (Shanghai, China). JC-1, propidium iodide (PI) and annexin V-FITC were purchased from Univ Biological Technology Co., Ltd. (Shanghai, China). Dichloromethane (DCM) and anhydrous dimethyl sulfoxide (DMSO) were purchased from Sinopharm Chemical Reagent Co., Ltd. (Shanghai, China).

### Cell culture

Non-small cell lung cancer cell line A549 and the PTX-resistant version of the same cell line, A549T, were kindly provided by Professor Yongzhuo Huang (Shanghai Institute of Materia Medica, Chinese Academy of Sciences). Both cell lines were grown in RPMI-1640 medium containing 10% fetal bovine serum and 100 μg/mL antibiotics (streptomycin and penicillin) at 37 °C in a humidified incubator with 5% CO_2_.

### Synthesis of the PTX-LMWP conjugate

#### Synthesis of PTX-SA

**Scheme 1.**
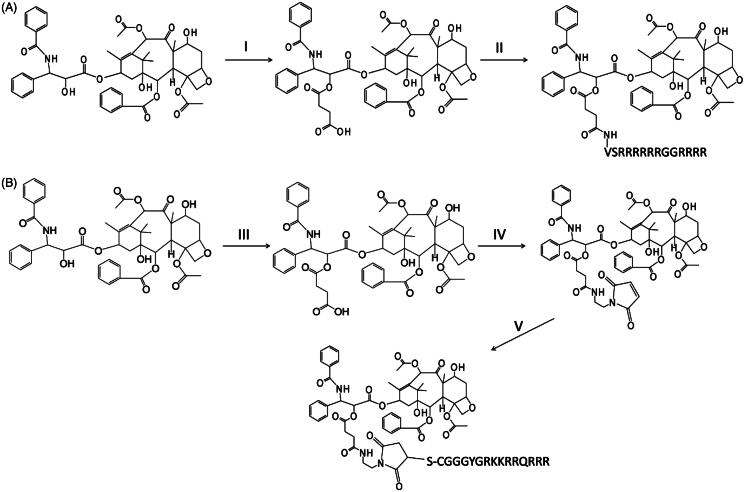
Reaction schemes for the syntheses of PTX-CPPs. (A) The synthesis of PTX-LMWP. (B) The synthesis of PTX-TAT. (I) Succinic acid, DIPEA, DCM, RT, 12 h. (II) EDC, NHS, DIPEA, LMWP, RT, DMSO, 24 h. (III) Succinic anhydride, DIPEA, DCM, RT, 12 h. (IV) EDC, NHS, DIPEA, N-(2-aminoethyl) maleimide, DMSO, RT, 12 h. (V) TAT, DMSO, RT, 1 h.

Succinic acid (SA) has a bis-carboxylic acid moiety and can react with the hydroxyl group in PTX on an equimolar basis to form a PTX-SA conjugate, which can be further linked with the primary amino group of LMWP via its free carboxyl group. Briefly, PTX (100 mg, 0.117 mmol) and succinic acid (20 mg, 0.169 mmol) were dissolved in 20 mL of anhydrous DCM. DIPEA (100 μL) was then added to the mixture as a catalyst. The reaction was performed with continuous stirring for 12 h at room temperature. As examined by thin layer chromatography (10:90, methanol:chloroform, v/v), PTX completely disappeared from the reaction mixture, and a new compound, PTX-SA, was formed. The resulting solution was condensed with a rotary evaporator. The residue was purified using a silica gel column and eluted with a chloroform–methanol mixture (10:0.5, v/v) to afford pure PTX-SA, which was then dried using a rotary evaporator (Scheme 1). ^1^H NMR (CDCl_3_, 400 MHz): *δ*_PPM_ 1.154 (s, 3H), 1.246 (s, 3H), 1.262–1.303 (m, 1H), 1.698 (s, 3H), 1.810–1.910 (m, 1H), 1.932 (s, 3H), 2.153–2.214 (m, 1H), 2.233 (s, 3H), 2.334–2.431 (m, 2H), 2.458 (s, 3H), 2.512–2.635 (m, 4H), 2.653–2.804 (m, 3H), 3.824 (d, 1H, *J* = 7.2 Hz), 4.183–4.307 (AB, 2H, *J* = 42.8 Hz), 4.398–4.496 (m, 1H), 4.995 (d, 1H, *J* = 7.6 Hz), 5.551 (d, 1H), 5.708 (d, 1H, *J* = 7.2 Hz), 5.975–6.040 (m, 1H), 6.254 (t, 1H, *J* = 8.4 Hz), 6.313 (s, 1H), 7.101 (d, 1H, *J* = 9.2 Hz), 7.330–7.378 (m, 1H), 7.395–7.475 (m, 6H), 7.483–7.560 (m, 3H), 7.593–7.663 (m, 1H), 7.781 (d, 2H, *J* = 7.2 Hz), 8.156 (d, 2H, *J* = 6.8 Hz). [M + H]^+^ HRMS (Q-TOF) *m*/*z*: 954.3523 (Supplementary material, Figures S1 and S2).

#### Synthesis of PTX-LMWP

Conjugation of SA with PTX resulted in the formation of a monocarboxylic acid group, which was further connected to the primary amino group in LMWP. Briefly, PTX-SA (100 mg, 0.104 mmol), EDC (200 mg, 1.043 mmol) and NHS (24 mg, 0.208 mmol) were dissolved in 5 mL of anhydrous DMSO and stirred at room temperature for 2 h. Then, LMWP and DIPEA (50 μL) were added, and the reaction solution was maintained under stirring at room temperature. The molar ratio of LMWP to PTX-SA was maintained at 1:1. The reaction was supervised by ultra-performance liquid chromatography (UPLC, BEH C18 column, 1.7 μm, 2.1 × 50 mm). The flow rate was 0.3 mL/min, with the mobile phase starting at 90% solvent A (0.1% TFA in water) and 10% solvent B (0.1% TFA in acetonitrile) at 0 min and transitioning to 40% solvent A and 60% solvent B at 10 min. The retention time for PTX-LMWP was 6.6 min. After 24 h, 1 mL of phosphate-buffered saline (PBS, pH = 5.0) was added to stop the reaction, and the product was purified by semi-preparative high performance liquid chromatography (HPLC; Eclipse XDB-C18, 9.4 × 250 mm). The flow rate was 3 mL/min, with the mobile phase starting at 70% solvent A and 30% solvent B (0 min) and transitioning to 10% solvent A and 90% solvent B (30 min). Solvent A and solvent B were the same as for the analytical UPLC method. Finally, the solution collected from semi-preparative HPLC was lyophilized for 2 d (Scheme 1). ^1^H NMR (D_2_O and DMSO-d6, 600 MHz): *δ*_PPM_ 0.752–0.863 (m, 6H), 0.929–1.015 (m, 6H), 1.399–1.582 (m, 30H), 1.608–1.775 (m, 16H), 1.859–1.970 (m, 3H), 2.084 (s, 3H), 2.153–2.222 (m, 5H), 2.264–2.470 (m, 6H), 2.538–2.633 (m, 4H), 3.136–3.370 (m, 4H), 3.510–3.671 (m, 5H), 3.717 (t, 3H, *J* = 16.8 Hz), 3.850–3.900 (m, 2H), 3.955–4.129 (m,7H), 4.134–4.314 (m,12H), 4.892 (d, 2H, *J* = 9.6 Hz), 5.273 (t, 2H, *J* = 9.6 Hz), 5.388 (d, 2H, *J* = 7.2 Hz), 5.437–5.487 (m, 2H), 5.750–5.839 (m, 2H), 7.160 (t, 1H, *J* = 7.2 Hz), 7.369–7.462 (m, 4H), 7.483 (t, 2H, *J* = 7.2 Hz), 7.536–7.565 (m, 1H), 7.626–7.686 (m, 2H), 7.737 (t, 1H, *J* = 7.2 Hz),7.816 (d, 2H, *J* = 7.2 Hz), 7.951 (d, 2H, *J* = 7.2 Hz). [M + H]^+^ HRMS (MALDI-TOF) *m*/*z*: 2816.4023 (Supplementary material, Figures S3, S4 and S5).

### Synthesis of the PTX-TAT conjugate

#### Synthesis of PTX-SA-MAL

There are three primary amino groups in TAT, including two lysine amino acid residues in its sequence. If TAT was conjugated with PTX through the reaction of a primary amino group and a carboxylic acid group in the same way as LMWP, a complicated mixture including at least three types of PTX-TAT conjugates would be generated because of the random reaction of PTX-SA with one or more of the three primary amino groups in TAT. In addition, because the lysine amino acid residues play a crucial role in the transduction function of TAT (Park et al., 2002), reacting the primary amino groups of the lysines would decrease the ability of TAT to translocate into cells. To avoid the occurrence of these issues, *N*-(2-aminoethyl) maleimide trifluoroacetate salt, a heterobifunctional cross-linker, was first reacted with PTX-SA. Briefly, PTX-SA (100 mg, 0.104 mmol), EDC (200 mg, 1.043 mmol), and NHS (24 mg, 0.208 mmol) were dissolved in 5 mL of anhydrous acetonitrile and stirred at room temperature for 2 h. Then, N-(2-aminoethyl)maleimide trifluoroacetate salt (30 mg, 0.118 mmol, dissolved in 10 mL of PBS, pH = 7.0) was added. The resulting solution was stirred for 12 h. Then, acetonitrile was removed with a rotary evaporator, and DCM (10 mL) was added as an extractive solvent to remove excessive EDC and NHS. The water phase was abandoned, and the organic phase was concentrated with a rotary evaporator. The residue of the organic phase was purified with a silica gel column and eluted with a chloroform:ethyl acetate mixture (4:10, v/v) to obtain pure PTX-SA-MAL (Scheme 1). ^1^H NMR (DMSO-d6, 400 MHz): *δ*_PPM_ 0.968 (s, 3H), 0.998 (s, 3H), 1.212 (s, 1H), 1.416–1.449 (m, 1H), 1.470 (s, 3H), 1.534–1.667 (m, 1H), 1.739 (s, 3H), 1.758–1.816 (m, 1H), 2.080 (s, 3H), 2.209 (s, 3H), 2.230–2.340 (m, 3H), 2.512–2.624 (m, 2H), 3.055–3.158 (m, 2H), 3.390 (t, 2H, *J* = 6 Hz), 4.034–4.126 (m, 1H), 4.627 (s, 1H), 4.886 (d, 1H, *J* = 10 Hz), 4.926 (s, 1H), 5.308 (d, 1H, *J* = 9.2 Hz), 5.386 (d, 1H, *J* = 7.2 Hz), 5.495 (t, 1H, *J* = 8.4 Hz), 5.788 (t, 1H, *J* = 8.8 Hz), 6.264 (s, 1H), 6.977 (s, 2H), 7.132–7.188 (m, 1H), 7.389–7.446 (m, 4H), 7.446–7.511 (m, 2H), 7.517–7.574 (m, 1H), 7.620–7.684 (m, 2H), 7.692–7.753 (m, 1H), 7.833 (d, 2H, *J* = 6.8 Hz), 7,958 (d, 2H, *J* = 7.2 Hz), 8.000 (t, 1H, *J* = 6 Hz), 9.210 (d, 1H, *J* = 8.4 Hz). [M + H]^+^ HRMS (Q-TOF) m/z: 1076.4033 (Supplementary material, Figures S6 and S7).

#### Synthesis of PTX-TAT

Linking a heterobifunctional cross-linker to PTX-SA provided a maleimide group that could react with the thiol group of TAT. Briefly, PTX-SA-MAL (100 mg, 0.093 mmol) and TAT (200 mg, 0.109 mmol) were dissolved in a solution comprising 5 mL of acetonitrile and 10 mL of PBS (pH = 7.0). The solution was then stirred at room temperature for 1 h. The reaction was monitored by UPLC using the same column and analytical method as that used for PTX-LMWP. The retention time for PTX-TAT was 6.2 min. The solution was purified by semi-preparative HPLC, also using the same method as that used for PTX-LMWP. The collected semi-preparative HPLC elution was lyophilized immediately (Scheme 1). ^1^H NMR (D_2_O and DMSO-d6, 600 MHz): *δ*_PPM_ 0.946 (s, 3H), 0.959 (s, 3H), 1.206–1.375 (m, 7H), 1.445 (s, 3H), 1.471–1.614 (m, 18H), 1.625–1.830 (m, 14H), 1.837–1.928 (m, 2H), 2.064 (s, 3H),2.159 (s, 6H), 2.252–2.362 (m, 4H), 2.546–2.636 (m, 3H), 2.682–2.746, 2.896–2.959 (brs, 4H), 2.773 (t, 4H, *J* = 7.2Hz), 3.095–3.267 (m, 6H), 3.332–3.451 (m, 3H), 3.470–3.558 (m, 2H), 3.583–3.799 (m, 7H), 3.799–3.920 (AB, 3H), 3.941 (t, 1H, *J* = 6.0Hz), 3.986 (s, 2H), 4.021–4.092 (m, 2H), 4.102–4.230 (m, 8H), 4.864–4.929, 5.327–5.385 (AB, brs, 4H), 5.231–5.287, 5.385–5.444 (AB, brs, 4H), 5.711–5.806 (m, 2H), 6.212 (s, 1H), 6.619–6.704, 6.974–7.067 (AA‘BB’; 4H), 7.090–7.136 (m, 1H), 7.363–7.421 (m, 4H), 7.455 (t, 2H, *J* = 7.8 Hz), 7.536 (t, 1H, *J* = 7.2 Hz), 7.632 (t, 2H, *J* = 7.8 Hz), 7.719 (t, 1H, *J* = 7.2 Hz), 7.765 (d, 2H, *J* = 7.2 Hz), 7.923 (d, 2H, *J* = 7.8). [M + H]^+^ HRMS (MALDI-TOF) *m*/*z*: 2910.3787 (Supplementary material, Figures S8, S9 and S10).

### Cellular uptake assay

The cellular uptake experiment was implemented basing on the protocol designed by Meng with some modifications (Meng et al., 2011). Briefly, A549 and A549T cells were cultivated in chambered 24-well plates with 5% CO_2_ at 37 °C for 48 h. Then, the adherent cells were washed twice with PBS. Each of the three drugs was diluted in cell medium at concentrations of 10 μM and 20 μM and added to the chambers. The cells were incubated for 2 h and washed four times with PBS after incubation. After that, the cells were scraped from the wells and quantitatively transferred to Eppendorf centrifuge tubes. The resulting cell/saline suspensions were sonicated for 20 cycles of 2 s each with 2 s of rest between each cycle using a probe sonicator. The lysed cell solution was centrifuged at 7000 *g* for 30 min at 4 °C. Then, 100 μL of the supernatant was withdrawn carefully and tested with UPLC (BEH C18, 1.7 μm, 2.1 × 50 mm). The flow rate was 0.3 mL/min, and the mobile phase consisted of 55% solvent A (PBS, pH = 5) and 45% solvent B (acetonitrile). To further test the change in cellular uptake against incubation time, the drugs (20 μM) were added to the cells, and the cells were then incubated for different times, ranging from 15 min to 4 h. The other steps of the experiment were performed as described above.

### Cytotoxicity study

The inhibitory activities of the drug samples on cell growth were evaluated using a 3-(4,5-dimethylthiazol-2-yl)-2,5-diphenyltetrazolium bromide (MTT) colorimetric assay. A549 and A549T cells were harvested by trypsinization and resuspended at a concentration of 2 × 10^4^ cells/mL in fresh culture medium. Then, the cells were seeded at a density of 5000 cells per well in 96-well plates. After 48 h of incubation at 37 °C with 5% CO_2_, the culture medium was replaced with 200 mL of medium containing one of the three drug samples, PTX, PTX-LMWP, or PTX-TAT. The concentration of the drugs ranged from 0.1 μM to 100 μM. After 24 h of incubation, the drug-containing media were abandoned, and 20 μL of MTT (5 mg/mL) solution was added to each well. The plate was incubated for an additional 4 h, and then, 200 mL of DMSO was added to each well to dissolve any formed purple formazan crystals. The plates were vigorously shaken before measuring the relative color intensity. The absorbance at 570 nm of each well was measured with a plate reader.

### Cell apoptosis detection

Cell apoptosis was determined by an annexin V-FITC/PI assay. The externalization of phosphatidylserine, a result of early stage apoptosis, was detected by FITC-annexin V protein staining, and the membrane damage due to late-stage apoptosis was detected by the binding of PI to nuclear DNA. Briefly, 2 × 10^5^ A549 and A549T cells were plated in 6-well plates and incubated for 24 h. After exposure to the different drugs at a concentration of 10 μM for 48 h, the cells were harvested by trypsinization, washed in PBS twice and incubated in the dark for 10 min at room temperature in 500 μL of binding buffer containing annexin V-FITC (1 μg/mL) and PI (1 μg/mL). The cells were immediately analyzed by flow cytometry (Becton Dickinson, Franklin Lakes, NJ). The fluorescence intensity of annexin V-FITC was measured at an excitation/emission wavelength of 488/530 nm and that of PI was measured at an excitation/emission wavelength of 488/617 nm. Approximately 10 000 cells were analyzed in each of the samples. Each assay was repeated in triplicate.

### Mitochondrial membrane potential (Δψm) determination

Changes in the mitochondrial membrane potential (Δψm) were detected using JC-1 and analyzed with flow cytometry. Briefly, A549 and A549T cells were seeded at a density of 1 × 10^5^ cells/mL in 6-well plates and exposed to the drugs (10 μM) for 48 h. The control experiments were performed by adding only culture medium. Then, the cells were harvested by trypsinization, washed in PBS twice, incubated with JC-1 (5 mg/mL) at 37 °C for 10 min in the dark, and finally analyzed by flow cytometry (Becton Dickinson, Franklin Lakes, NJ). Each assay was repeated in triplicate. JC-1 accumulates in mitochondria that have a high membrane potential and then dimerizes due to the high local concentrations achieved. Dimerized JC-1 emits red fluorescence, while monomeric JC-1 emits green fluorescence. The results are expressed as a ratio of red to green fluorescence. High values indicate that the cells have an intact mitochondrial membrane potential, while low values suggest the cells are in the early stage of apoptosis.

### Cell-cycle analysis

To analyze the effect of the PTX-CPP conjugates on the cell cycle distribution, A549 and A549T cells were seeded at a density of 1 × 10^5^ cells/mL in 6-well plates and then exposed to 5 μM PTX, PTX-TAT, or PTX-LMWP solution for 48 h. After that, the cells were harvested by trypsinization, washed in PBS twice, and fixed in 70% cold ethanol for 24 h at 4 °C. The fixed cells were washed in cold PBS and then resuspended in 0.5 mL of PBS containing 50 μg/mL PI, 0.1% Triton-X-100, 0.1% sodium citrate, and 100 μg/mL RNase. After incubation at 4 °C in the dark for 30 min, the fluorescence-activated cells were sorted. The cellular DNA content was analyzed by flow cytometry (Becton Dickinson, Franklin Lakes, NJ). Each analysis contained at least 10 000 cells. The changes in cell distribution at each cell-cycle phase were observed, and the results are displayed in histograms. The percentages of cells in different cell cycle phases were then recorded. Each experiment was performed in triplicate, and the results were expressed as the mean ± SD.

### Antitumor efficacy

The protocol to evaluate the *in vivo* antitumor efficacy of the PTX-CPP conjugates was approved by the ethics committee of Fudan University. Four-week-old female BALB/c nude mice were purchased and housed in the Animal Care Facilities in the School of Pharmacy, Fudan University. BALB/c mice bearing subcutaneous tumors were used as an animal model for the evaluation of the anticancer activities of PTX-LMWP and PTX-TAT. The subcutaneous tumor xenograft model was established by inoculating 2 × 10^6^ A549 or A549T cells (in 100 μL of cell culture medium) into the subcutaneous tissue of the armpit of the right anterior limb (Gu et al., 2014). Tumor size was measured with Vernier calipers for the largest (length) and smallest (width) superficial visible diameters of the protruding tumor mass through the skin. Tumor volumes were calculated according to the following formula: volume = 0.52 × *W*^2^×*L*, where *W* and *L* represent the width and the length, respectively (Lee et al., 2011). The drugs were administered 2 weeks after tumor implantation, when the size of the tumors reached approximately 100–150 mm^3^. Test compounds included (I) PBS solution (control), (II) PTX (1000 μM), (III) PTX-LMWP (1000 μM), and (IV) PTX-TAT (1000 μM). Each experimental group contained six mice. The mice were treated (100 μL volume) three times over 8 d via peritumoral injection. Tumor volumes were measured at 2-d intervals. At the end of the experiment, the mice were sacrificed, and necropsies were performed. The tumors were removed, weighed and fixed with formalin.

## Results

### Cellular uptake assay

**Figure 1.**
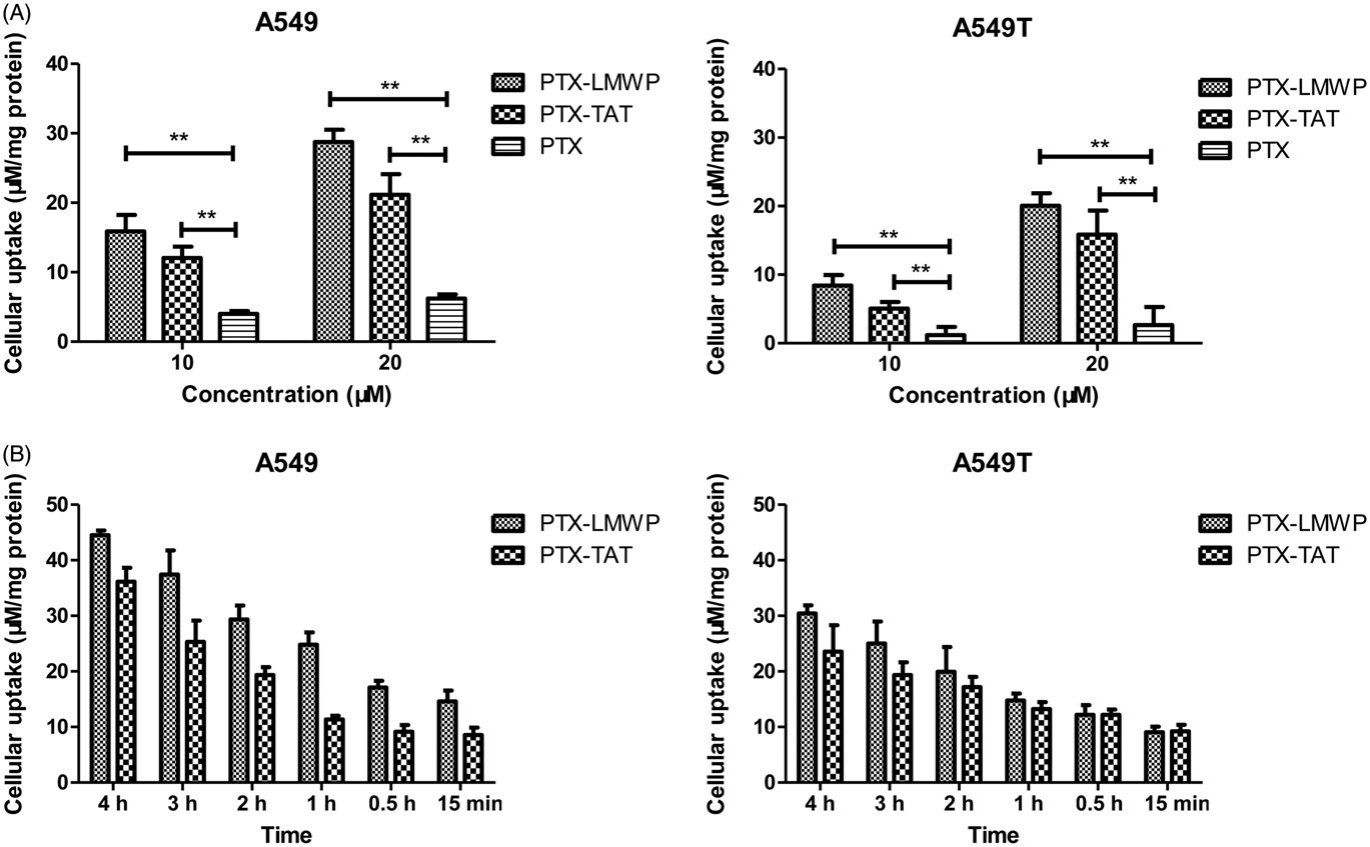
Intracellular drug accumulation of PTX and PTX-CPPs. (A) Cells were incubated with different concentrations of PTX and PTX-CPPs for 2 h. (B) Cells were incubated with 20 μM PTX and PTX-CPPs for different times. **p < 0.01.

The cellular uptake of PTX was evaluated in A549 and A549T cells. As shown in Figure 1(A), the results indicated that both A549 and A549T cells treated with PTX-CPPs had higher cellular uptake of PTX than cells incubated with PTX (*p* < 0.01), regardless of the concentration was 10 μM or 20 μM. As shown in Figure 1(B), the cellular uptake of PTX-CPPs increased with prolonged incubation time in both cell lines. The uptake of PTX-LMWP was stronger than that of PTX-TAT in A549 cells at all incubation times (*p* < 0.05), but this phenomenon was not observed in A549T cells.

### Cytotoxicity study

**Figure 2.**
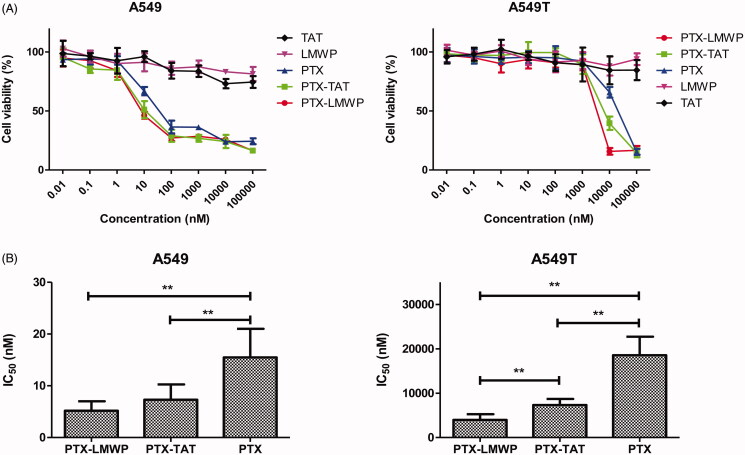
Viability of A549 and A549T cells treated with different concentrations of PTX and PTX-CPPs. (A) Cell viability. (B) IC_50_. **p < 0.01.

The cytotoxic efficiency of PTX and PTX-CPPs toward tumor cells was measured in A549 and A549T cells. The concentration of PTX and PTX-CPPs ranged from 0.01 nM to 100 000 nM. LMWP and TAT peptides were also selected in the same molar concentrations as PTX to test the toxicity of the CPPs. In MTT assay, all cells exposed to PTX/PTX-CPPs exhibited a typical dose-dependent curve. The viability of A549 cells was higher than 80% when the concentration of PTX was below 1 nM. When the concentrations of PTX and PTX-CPPs ranged from 100 nM to 100 000 nM, the cell viabilities decreased to 40%. As shown in Figure 2(A), from 1 nM to 100 nM, the survival curves of the cells treated with PTX-CPPs declined more sharply than that of the cells treated with PTX. In A549T cells, no effect on cell viability was observed in response to either PTX-CPPs or PTX when the concentrations were below 1000 nM because of the drug resistance of these tumor cells. Meanwhile, the cell viabilities of the PTX-CPP groups were notably decreased compared with those of the PTX groups when the concentration changed from 1000 nM to 100 000 nM. No harmful effect was observed in A549 or A549T cells treated with TAT or LMWP alone.

GraphPad Prism software (GraphPad Software, Inc., La Jolla, CA) was used to calculate the IC_50_ of PTX and the PTX-CPPs. As shown in Figure 2(B), in both A549 cells and A549T cells, the IC_50_ values of PTX-LMWP and PTX-TAT, which were 33.5% and 47.2% of PTX in A549 cells and 21.5% and 39.5% of PTX in A549T cells, respectively, were decreased significantly compared with that of PTX (*p* < 0.01). The observed IC_50_ value of PTX-LMWP was remarkably lower than that of PTX-TAT in A549T cells (*p* < 0.01), while this phenomenon was not observed in A549 cells.

### Cell apoptosis

**Figure 3.**
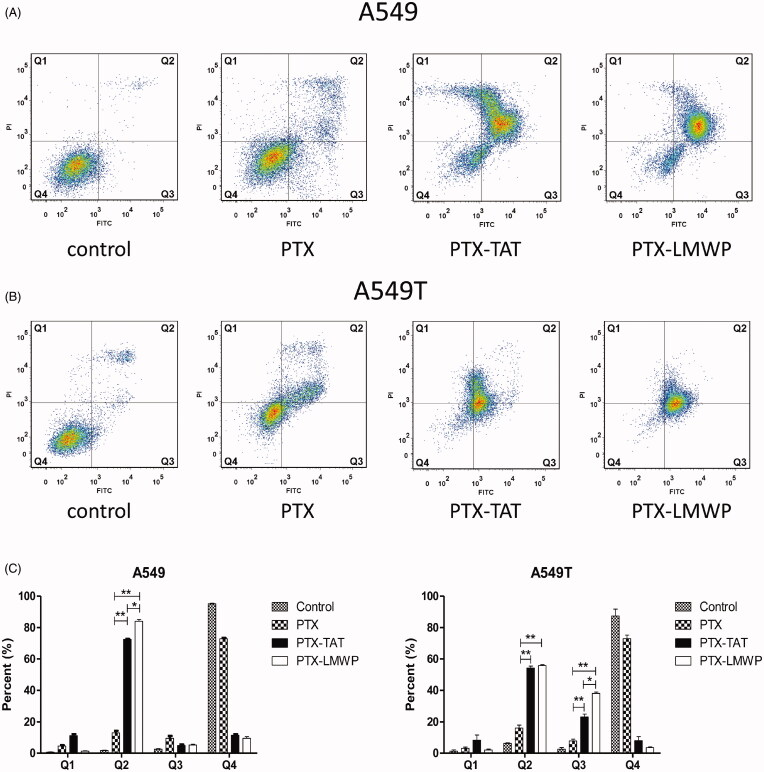
Cell apoptosis after incubating with 10 μM PTX or PTX-CPPs for 48 h. (A) Apoptosis of A549 cells. (B) Apoptosis of A549T cells. (C) Quantification of apoptosis data. *p < 0.05; **p < 0.01.

As shown in Figure 3(A,B), the Q1, Q2, Q3, and Q4 regions represent necrotic cells (PI+/annexin V−), late apoptotic cells (PI+/annexin V+), early apoptotic cells (PI−/annexin V+) and viable cells (PI−/annexin V−), respectively. The apoptosis results revealed that PTX-CPPs induced early apoptosis and/or late apoptosis in more tumor cells than PTX. From Figure 3(C), it can be seen that PTX induced late apoptosis in only 13.03% of A549 cells. After the treatment with PTX-TAT or PTX-LMWP, the percentage of late apoptotic A549 cells sharply increased to 72.57% or 84.03%, respectively (*p* < 0.01). PTX-LMWP had a better ability to induce late apoptosis in A549 cells than PTX-TAT (*p* < 0.05). In A549T cells, PTX-TAT and PTX-LMWP induced late apoptosis in 54.20% and 55.75% of cells, respectively, while PTX only induced late apoptosis in 16.10% of cells (*p* < 0.01). The percentages of early apoptotic A549T cells induced by PTX-TAT and PTX-LMWP were 23.15% and 38.10%, respectively, both of which were significantly higher than that of PTX (*p* < 0.01). Obviously, the effect of PTX-LMWP on inducing early apoptosis in A549T cells was stronger than that of PTX-TAT (*p* < 0.05).

### Mitochondrial membrane potential (Δψm)

**Figure 4.**
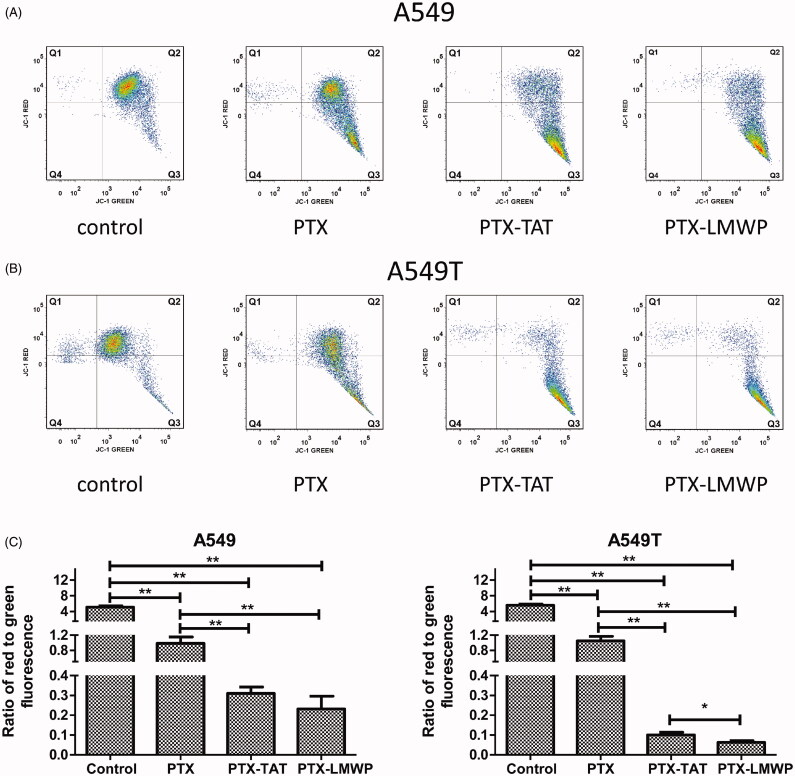
JC-1 forms and Δψm values of A549 and A549T cells. All cells were exposed to drugs at 10 μM for 48 h. (A) The distribution of different JC-1 forms in A549 cells. (B) The distribution of different JC-1 forms in A549T cells. (C) Ratio of red to green fluorescence in A549 and A549T cells. *p < 0.05; **p < 0.01.

The effects of PTX and PTX-CPPs on the mitochondrial membrane potential (Δψm) in A549 cells and A549T cells are shown in Figure 4(A,B). The *x*-axis and the *y*-axis display the JC-1 monolayer signals and JC-1 aggregate signals, respectively. Q1 + Q2 and Q3 + Q4 represent the aggregate form and monomeric form of JC-1, respectively. The results indicated that PTX and PTX-CPPs significantly reduced the Δψm in both A549 and A549T cells compared with the control cells (*p* < 0.01). PTX-CPPs induced more severe collapse of the Δψm than PTX in both cell lines (*p* < 0.01). In addition, in A549T cells, treatment with PTX-LMWP resulted in significant breakdown of the Δψm compared with the PTX-TAT treatment (*p* < 0.05). However, the same result was not observed in A549 cells.

### Cell-cycle analysis

**Figure 5.**
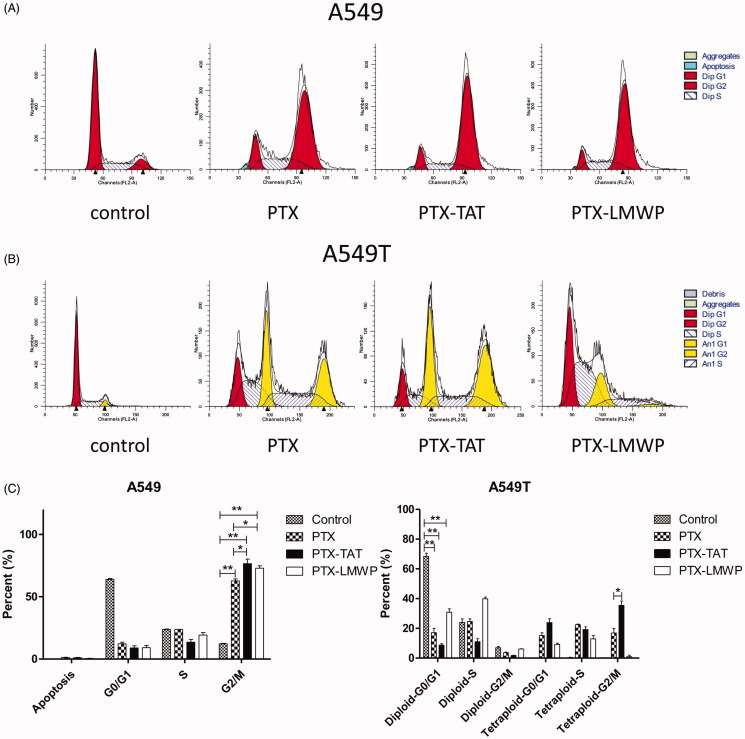
Cell-cycle profiles of A549 and A549T cells treated with 5 μM PTX or PTX-CPPs. (A) Cell-cycle distribution of A549 cells. (B) Cell-cycle distribution of A549T cells. (C) Quantification of cell cycle data. *p < 0.05; **p < 0.01.

The effects of PTX and PTX-CPPs on the cell-cycle distribution of A549 and A549T cells were displayed in Figure 5. In A549 cells, exposure to PTX and PTX-CPPs substantially increased the percentages of cells in G2/M phase cell-cycle arrest compared with the control cells (*p* < 0.01), and significantly higher percentages of G2/M phase cells were observed in PTX-CPP-treated cells than the cells treated with PTX (*p* < 0.05). In A549T cells, the fitting result showed that a large number of A549T cells were induced into tetraploids with exposure to PTX and PTX-TAT, and PTX-TAT-treated A549T cells showed higher tetraploid-G2/M phase arrest than those incubated with PTX (*p* < 0.05). In contrast, A549T cells treated with PTX-LMWP demonstrated limited generation of tetraploids but a large number of cells in diploid-S phase cell-cycle arrest. Compared with the control cells, the percentages of diploid-G0/G1 phase A549T cells in the PTX and PTX-CPP groups were extremely reduced (*p* < 0.01), which was similar to the results in A549 cells.

### Antitumor efficacy

**Figure 6.**
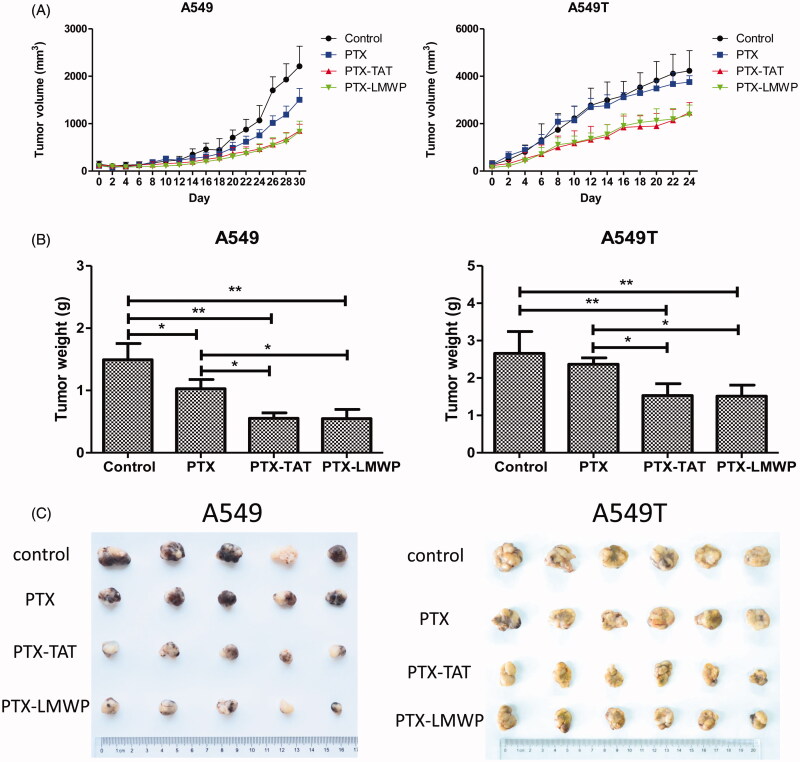
Anticancer efficacy of the drugs on tumor cell xenografts in female nude mice. (A) Tumor volume profiles. (B) Weights of tumors from mice after 24 or 30 d. (C) Photos of tumors separated from mice. *p < 0.05; **p < 0.01.

A549 and A549T tumor cells xenografted into nude mice were used for the *in vivo* efficacy study. As shown in Figure 6, A549 tumor growth in mice treated with PTX-CPPs was significantly suppressed, and the size of tumors reduced continuously compared with the control (*p* < 0.05) from 20 to 30 d. PTX treatment also had a significant antitumor effect in A549 tumor-bearing mice after 26 d (*p* < 0.05). Furthermore, PTX-TAT and PTX-LMWP produced more convincing growth inhibition of A549 tumors than PTX in nude mice (*p* < 0.05). The volume of tumor was decreased approximately 1.51–1.77-fold and 1.45–1.80-fold, respectively, from 22 to 30 d. PTX did not show satisfactory antitumor efficacy in A549T-tumor bearing mice, and there was no significant difference in the tumor volumes between the control and PTX groups. PTX-TAT and PTX-LMWP still had a considerable antitumor effect compared with the control or PTX (*p* < 0.05) after 6 d and displayed 1.74–1.52-fold and 1.74–1.58-fold reductions, respectively, in tumor volume from 6 to 24 d compared with the PTX group. After 24 or 30 d, all tumor-bearing mice were sacrificed. The tumors were dissected and weighed. The tumor inhibition rates (%) in A549 tumor-bearing mice receiving PTX-LMWP and PTX-TAT were 62.92% and 63.32%, respectively. Both showed significant inhibition (*p* < 0.05) compared with the 31.06% inhibition rate of PTX. Meanwhile, the inhibition rates (%) in A549T tumor-bearing mice injected with PTX-TAT and PTX-LMWP were 42.47% and 43.13%, respectively. Compared with the inhibition rate in the group treated with PTX, which was only 11.0%, the inhibitory abilities of PTX-LMWP and PTX-TAT were a significant improvement (*p* < 0.05). However, no significant difference was observed between the inhibitory effects of PTX-TAT and PTX-LMWP.

## Discussion

Here, we have developed two novel conjugates, PTX-LMWP and PTX-TAT, to enhance the cellular uptake and antitumor efficacy of PTX, especially in drug-resistant tumor cells. To synthesize PTX-LMWP, a two-step procedure was used starting with 2′-hemisuccinylation of PTX through a previously reported reaction (Safavy et al., 2004; Khandare et al. 2006; Gao et al., 2009). Conjugation of PTX-SA to the LMWP peptide was performed using a previously described NHS/EDC method (Pan et al., 2016). However, direct reaction of PTX-SA with TAT would produce a complicated reaction mixture, including at least three forms of PTX-TAT conjugates, due to the random reaction of PTX-SA with the three primary amino groups of TAT. Therefore, many previous reports utilized a heterobifunctional cross-linker to synthesize TAT conjugates (Christie et al., 2004; Wei et al., 2009; van Bracht et al., 2014; Li et al., 2015). In this article, we chose a simple procedure of covalently bonding PTX-SA to N-(2-aminoethyl) maleimide using the NHS/EDC method to form PTX-SA-MAL, and then TAT was reacted with PTX-SA-MAL to form PTX-TAT conjugate.

The results of cellular uptake demonstrated that PTX-CPPs could enhance the accumulation of PTX in A549 and A549T cells compared with PTX, which confirm that CCPs can facilitate the uptake of cargo (Nakase et al., 2012). The uptake mechanism of CPPs has been deeply researched but still remained elusive (Guo et al., 2016). It is also unknown whether CPPs enter cells with or without the mediation of specific receptors (Farkhani et al., 2014). Nonetheless, it is a common consensus (Fonseca et al., 2009) that energy-dependent endocytosis and energy-independent direct translocation are two major cellular uptake mechanisms of CPPs. Small molecules linked with CPPs can enter cells quickly via direct translocation. In contrary, uptake of large cargos attached to CPPs may due to the energy-dependent endocytosis with slow rates. The direct translocation contains several different models (Guo et al., 2016) such as transient pore formation, carpet-like model, membrane-thinning model, and transitory membrane structure formation. The common features of these models are that CPPs bind to the plasma membrane via electrostatic interaction first, and then CPPs induce membrane destabilization or inverted micelle inside the lipid bilayer, leading to the uptake of CPPs. Previous research (Song et al., 2015) proved that TAT peptide could induce the formation of transient natural pore in membrane. Kawamoto (Kawamoto et al., 2011) illustrated that arginine-rich peptides permeated the plasma membrane via the formation of inverted micelle. LMWP peptide is an arginine-rich peptide. Based on the literatures and considering the small molecular weight of PTX, we can speculate that PTX-TAT and PTX-LMWP may enter the tumor cells via energy-independent direct translocation pathway. The uptake of PTX-TAT and PTX-LMWP increased without obviously saturate phenomenon when the incubation time was prolonged. The results suggest that the uptake of both conjugates may be mediated via energy-independent pathway. The uptake results of two conjugates are coincidence with our speculation. Efflux pump is a promiscuous transmembrane protein, thus PTX-CPPs can easily bypass the efflux pump via the transient pore or inverted micelle in membrane, which may be the reason that PTX-CPPs conjugates can overcome the drug resistance of A549T cells.

Apoptosis is a kind of programed cell death. The decrease of mitochondrial membrane potential is often a symbol of early apoptosis (Jiang et al., 2015). As a cationic dye, JC-1 can bind with polarized mitochondria in healthy cells and emit red fluorescence. Whereas in apoptotic cells, it can disperse from depolarized mitochondria to cytosol and emit green fluorescence. Thus, the disruption of mitochondrial membrane potential (Δψm) can be indicated by a switch from red to green fluorescence. According to previous researches (Pawar et al., 2014; Jelinek et al., 2015), PTX has been shown to activate the mitochondrial pathway of apoptosis. In this study, we observed significant reductions of the Δψm in A549 and A549T cells after application of PTX and PTX-CPPs. These data suggest that PTX-CPPs also induce cell apoptosis through mitochondrial pathway. PTX-CPPs displayed enhanced cell toxicity, greater apoptosis, and a greater reduction in the Δψm in both sensitive and drug-resistant cells compared with PTX, and this result may be ascribed to the extremely elevated accumulation of PTX-CPPs in cells. It is interesting that in A549 cells, PTX-LMWP and PTX-TAT exhibited different degrees of cellular uptake but manifested similar cell toxicities and effects on the Δψm, which indicates that the increased uptake of PTX-LMWP is not sufficient to exert a more considerable antitumor efficacy. In contrast, there was no significant difference between the cellular uptake of PTX-LMWP and PTX-TAT in A549T cells, while the cell toxicity, the effects on apoptosis and the Δψm of the two conjugates were quite different, indicating that the diverse anti-drug resistance mechanisms of PTX-TAT and PTX-LMWP may influence their antitumor efficiency in A549T cells.

The cell-cycle assessment showed that PTX-CPPs could induce more G2/M phase arrest compared with PTX and the control. This phenomenon may be attributed to the increased accumulation of PTX in A549 cells caused by LMWP and TAT. After treatment with PTX and PTX-CPPs, a large number of tetraploids were found in A549T cells. As is known, PTX can stabilize microtubules (Tian et al., 2014), hinder normal kinetochore-microtubule attachment and activate spindle assembly checkpoint, and subsequently lead to mitotic arrest and cell death (Ganem et al., 2007). However, after going through a process known as mitotic slippage, some cells are induced into apoptosis, while others can slowly recover from the arrest of the spindle assembly checkpoint and re-enter the G1 phase as tetraploids, thus escape the apoptosis induced by PTX (Flores et al., 2011; Topham & Taylor, 2013; Chi et al., 2014). Many studies revealed that polyploid cancer cells, especially tetraploids, displayed clear correlation with drug resistance (Puig et al., 2008; Ogden et al., 2015; Zhou et al., 2015). It is reasonable that A549T cells, as a drug-resistant cancer cell line, experienced an apparent tetraploid mitotic process after treatment with PTX and PTX-CPPs. Interestingly, PTX- and PTX-TAT-treated A549T cells had obvious tetraploid-G2/M phase arrest, but PTX-LMWP mostly induced diploid-G0/G1 and diploid-S phase arrest. Although the reason is still unclear, the phenomenon illustrates that PTX-LMWP has a different mechanism of anti-drug resistance with PTX and PTX-TAT. Considering tetraploid morphology is strongly associated with drug resistance, the small percentage of cells exhibiting tetraploid mitotic phase arrest indicates that PTX-LMWP is powerful in overcoming the drug resistance of A549T cells.

The *in vivo* antitumor assay showed that PTX-CPPs had stronger inhibitory effects on tumor volume and tumor weight than the control and PTX in both cell lines, consistent with the cell toxicity, cell apoptosis, and Δψm results. The result confirms that PTX-CPPs are more effective in slowing the growth of solid tumors, particularly drug-resistant tumors. However, there was no difference between PTX-TAT and PTX-LMWP in the inhibition of A549T tumors, which does not agree with the results of *in vitro* studies. Considering that all *in vitro* experiments were tested in cells with a single membrane, PTX-LMWP may have a higher capacity to penetrate single-cell membranes and induce apoptosis or death compared with PTX-TAT. However, the xenografted tumors in the nude mice recruited dense tumor tissues, which were likely much harder to be penetrated than a single cell *in vitro* (Lee et al., 2011). Thus, PTX-LMWP may not have sufficient ability to permeate solid A549T tumors more deeply than PTX-TAT. However, PTX-LMWP still showed a significant antitumor effect in both sensitive and drug-resistant tumors *in vivo*, and the effects of PTX-LMWP and PTX-TAT were both much stronger than that of PTX.

## Conclusion

In summary, two new conjugates, PTX-TAT and PTX-LMWP, were synthesized successfully. Both conjugates were more efficient in permeating tumor cells and inducing cell toxicity and apoptosis in both sensitive and drug-resistant lung cancer cells than PTX. Cell-cycle analysis showed that PTX-CPPs induced more G2/M phase cell-cycle arrest in A549 cells. With the emergence of tetraploid A549T cells, the cell-cycle distribution of A549T cells was very different from that of A549 cells. This result suggests that PTX-LMWP has a different mechanism of influencing mitosis in drug-resistant lung tumor cells than PTX and PTX-TAT. The *in vivo* antitumor efficacy study confirms that PTX-CPPs could significantly inhibit the growth of both sensitive and drug-resistant tumors. Compared with PTX-TAT, PTX-LMWP exhibited much stronger effects on cell toxicity and apoptosis. The results of this study highlight the potential of PTX-CPPs, especially PTX-LMWP, which are promising for clinical application in the treatment of drug-resistant cancers.

## Supplementary Material

Supplemental_material.docx

## Notes

**Supplementary material available online**

## References

[CIT0001] Chen Z, Zhang P, Cheetham AG, et al. (2014). Controlled release of free doxorubicin from peptide-drug conjugates by drug loading. J Control Release 191:123–30 24892976 10.1016/j.jconrel.2014.05.051

[CIT0002] Chi KN, Higano C, Reeves J, et al. (2014). A randomized phase III study comparing first line docetaxel and prednisone (DP) to DP plus custirsen in men with metastatic castration resistant prostate cancer. Ann Oncol 25:255–79

[CIT0003] Christie RJ, Findley DJ, Grainger DW. (2004). Design and synthesis of a new polymer drug delivery conjugate. Biomed Sci Instrum 40:136–41 15133948

[CIT0004] De Hoon JP, Veeck J, Vriens BE, et al. (2012). Taxane resistance in breast cancer: a closed HER2 circuit? Biochim Biophys Acta 1825:197–206 22280939 10.1016/j.bbcan.2012.01.001

[CIT0005] De La Torre BG, Hornillos V, Luque-Ortega JR, et al. (2014). A BODIPY-embedding miltefosine analog linked to cell-penetrating Tat(48–60) peptide favors intracellular delivery and visualization of the antiparasitic drug. Amino Acids 46:1047–58 24445871 10.1007/s00726-013-1661-3

[CIT0006] Farkhani SM, Valizadeh A, Karami H, et al. (2014). Cell penetrating peptides: efficient vectors for delivery of nanoparticles, nanocarriers, therapeutic and diagnostic molecules. Peptides 57:78–94 24795041 10.1016/j.peptides.2014.04.015

[CIT0007] Ferrara R, Pilotto S, Peretti U, et al. (2016). Tubulin inhibitors in non-small cell lung cancer: looking back and forward. Expert Opin Pharmacother 17:1113–29 26898217 10.1517/14656566.2016.1157581

[CIT0008] Flores ML, Castilla C, Avila R, et al. (2011). Paclitaxel sensitivity of breast cancer cells requires efficient mitotic arrest and disruption of Bcl-xL/Bak interaction. Breast Cancer Res Treat 133:917–28 22076480 10.1007/s10549-011-1864-9

[CIT0009] Fonseca SB, Pereira MP, Kelley SO. (2009). Recent advances in the use of cell-penetrating peptides for medical and biological applications. Adv Drug Deliv Rev 61:953–64 19538995 10.1016/j.addr.2009.06.001

[CIT0010] Galletti E, Magnani M, Renzulli ML, Botta M. (2007). Paclitaxel and docetaxel resistance: molecular mechanisms and development of new generation taxanes. ChemMedChem 2:920–42 17530726 10.1002/cmdc.200600308

[CIT0011] Ganem NJ, Storchova Z, Pellman D. (2007). Tetraploidy, aneuploidy and cancer. Curr Opin Genet Dev 17:157–62 17324569 10.1016/j.gde.2007.02.011

[CIT0012] Gao Y, Kuang Y, Guo Z, et al. (2009). Enzyme-instructed molecular self-assembly confers nanofibers and a supramolecular hydrogel of taxol derivative. J Am Chem Soc 131:13576–7 19731909 10.1021/ja904411z

[CIT0013] Gu G, Hu Q, Feng X, et al. (2014). PEG-PLA nanoparticles modified with APTEDB peptide for enhanced anti-angiogenic and anti-glioma therapy. Biomaterials 35:8215–26 24974009 10.1016/j.biomaterials.2014.06.022

[CIT0014] Guo Z, Peng H, Kang J, Sun D. (2016). Cell-penetrating peptides: possible transduction mechanisms and therapeutic applications. Biomed Rep 4:528–34 27123243 10.3892/br.2016.639 PMC4840506

[CIT0015] Han M, Vakili MR, Soleymani Abyaneh H, et al. (2014). Mitochondrial delivery of doxorubicin via triphenylphosphine modification for overcoming drug resistance in MDA-MB-435/DOX cells. Mol Pharm 11:2640–9 24811541 10.1021/mp500038g

[CIT0016] Jelinek M, Balusikova K, Schmiedlova M, et al. (2015). The role of individual caspases in cell death induction by taxanes in breast cancer cells. Cancer Cell Int 15:8 25685064 10.1186/s12935-015-0155-7 PMC4329194

[CIT0017] Jiang L, Li L, He X, et al. (2015). Overcoming drug-resistant lung cancer by paclitaxel loaded dual-functional liposomes with mitochondria targeting and pH-response. Biomaterials 52:126–39 25818419 10.1016/j.biomaterials.2015.02.004

[CIT0018] Kawamoto S, Takasu M, Miyakawa T, et al. (2011). Inverted micelle formation of cell-penetrating peptide studied by coarse-grained simulation: importance of attractive force between cell-penetrating peptides and lipid head group. J Chem Phys 134:095103 21385001 10.1063/1.3555531

[CIT0019] Khandare JJ, Jayant S, Singh A, et al. (2006). Dendrimer versus linear conjugate: influence of polymeric architecture on the delivery and anticancer effect of paclitaxel. Bioconjug Chem 17:1464–72 17105225 10.1021/bc060240p

[CIT0020] Kibria G, Hatakeyama H, Harashima H. (2014). Cancer multidrug resistance: mechanisms involved and strategies for circumvention using a drug delivery system. Arch Pharm Res 37:4–15 24272889 10.1007/s12272-013-0276-2

[CIT0021] Lee J-Y, Choi Y-S, Suh J-S, et al. (2011). Cell-penetrating chitosan/doxorubicin/TAT conjugates for efficient cancer therapy. Int J Cancer 128:2470–80 20669230 10.1002/ijc.25578

[CIT0022] Li F, Wang Z, Huang Y, et al. (2015). Delivery of PUMA apoptosis gene using polyethyleneimine-SMCC-TAT/DNA nanoparticles: biophysical characterization and in vitro transfection into malignant melanoma cells. J Biomed Nanotechnol 11:1776–82 26502640 10.1166/jbn.2015.2151

[CIT0023] Li J, Huang P, Chang L, et al. (2013). Tumor targeting and pH-responsive polyelectrolyte complex nanoparticles based on hyaluronic acid-paclitaxel conjugates and Chitosan for oral delivery of paclitaxel. Macromol Res 21:1331–7

[CIT0024] Liang JF, Yang VC. (2005). Synthesis of doxorubicin-peptide conjugate with multidrug resistant tumor cell killing activity. Bioorg Med Chem Lett 15:5071–5 16168650 10.1016/j.bmcl.2005.07.087

[CIT0025] Liang L, Lin SW, Dai W, et al. (2012). Novel cathepsin B-sensitive paclitaxel conjugate: higher water solubility, better efficacy and lower toxicity. J Control Release 160:618–29 22410114 10.1016/j.jconrel.2012.02.020

[CIT0026] Luo S, Gu Y, Zhang Y, et al. (2015). Precise ratiometric control of dual drugs through a single macromolecule for combination therapy. Mol Pharm 12:2318–27 26035636 10.1021/mp500867g

[CIT0027] Luqmani YA. (2005). Mechanisms of drug resistance in cancer chemotherapy. Med Princ Pract 14:35–48 10.1159/000086183 16103712

[CIT0028] Meng S, Su B, Li W, et al. (2011). Integrin-targeted paclitaxel nanoliposomes for tumor therapy. Med Oncol 28:1180–7 20645030 10.1007/s12032-010-9621-1

[CIT0029] Minko T, Rodriguez-Rodriguez L, Pozharov V. (2013). Nanotechnology approaches for personalized treatment of multidrug resistant cancers. Adv Drug Deliv Rev 65:1880–95 24120655 10.1016/j.addr.2013.09.017

[CIT0030] Murakami M, Ernsting MJ, Undzys E, et al. (2013). Docetaxel conjugate nanoparticles that target alpha-smooth muscle actin-expressing stromal cells suppress breast cancer metastasis. Cancer Res 73:4862–71 23907638 10.1158/0008-5472.CAN-13-0062

[CIT0031] Nakase I, Konishi Y, Ueda M, et al. (2012). Accumulation of arginine-rich cell-penetrating peptides in tumors and the potential for anticancer drug delivery *in vivo*. J Control Release 159:181–8 22285548 10.1016/j.jconrel.2012.01.016

[CIT0032] Nicholson DW. (2000). From bench to clinic with apoptosis-based therapeutic agents. Nature 407:810–16 11048733 10.1038/35037747

[CIT0033] Ogden A, Rida PC, Knudsen BS, et al. (2015). Docetaxel-induced polyploidization may underlie chemoresistance and disease relapse. Cancer Lett 367:89–92 26185000 10.1016/j.canlet.2015.06.025 PMC4813805

[CIT0034] Pan Z-Z, Wang H-Y, Zhang M, et al. (2016). Nuclear-targeting TAT-PEG-Asp8-doxorubicin polymeric nanoassembly to overcome drug-resistant colon cancer. Acta Pharmacol Sin 37:1110–120 27292613 10.1038/aps.2016.48 PMC4973383

[CIT0035] Park J, Ryu J, Kim K-A, et al. (2002). Mutational analysis of a human immunodeficiency virus type 1 Tat protein transduction domain which is required for delivery of an exogenous protein into mammalian cells. J Gen Virol 83:1173–81 11961273 10.1099/0022-1317-83-5-1173

[CIT0036] Patel NR, Pattni BS, Abouzeid AH, Torchilin VP. (2013). Nanopreparations to overcome multidrug resistance in cancer. Adv Drug Deliv Rev 65:1748–62 23973912 10.1016/j.addr.2013.08.004 PMC3840079

[CIT0037] Patil Y, Sadhukha T, Ma L, Panyam J. (2009). Nanoparticle-mediated simultaneous and targeted delivery of paclitaxel and tariquidar overcomes tumor drug resistance. J Control Release 136:21–9 19331851 10.1016/j.jconrel.2009.01.021

[CIT0038] Pawar VK, Panchal SB, Singh Y, et al. (2014). Immunotherapeutic vitamin E nanoemulsion synergies the antiproliferative activity of paclitaxel in breast cancer cells via modulating Th1 and Th2 immune response. J Control Release 196:295–306 25459427 10.1016/j.jconrel.2014.10.010

[CIT0039] Puig PE, Guilly MN, Bouchot A, et al. (2008). Tumor cells can escape DNA-damaging cisplatin through DNA endoreduplication and reversible polyploidy. Cell Biol Int 32:1031–43 18550395 10.1016/j.cellbi.2008.04.021

[CIT0040] Pusztai L, Wagner P, Ibrahim N, et al. (2005). Phase II study of tariquidar, a selective P-glycoprotein inhibitor, in patients with chemotherapy-resistant, advanced breast carcinoma. Cancer 104:682–91 15986399 10.1002/cncr.21227

[CIT0041] Rasco DW, Yan J, Xie Y, et al. (2010). Looking beyond surveillance, epidemiology and end results: patterns of chemotherapy administration for advanced non-small cell lung cancer in a contemporary, diverse population. J Thorac Oncol 5:1529–35 20631635 10.1097/JTO.0b013e3181e9a00f PMC3466589

[CIT0042] Regina A, Demeule M, Che C, et al. (2008). Antitumour activity of ANG1005, a conjugate between paclitaxel and the new brain delivery vector Angiopep-2. Br J Pharmacol 155:185–97 18574456 10.1038/bjp.2008.260 PMC2538693

[CIT0043] Safavy A, Georg GI, Velde DV, et al. (2004). Site-specifically traced drug release and biodistribution of a paclitaxel-antibody conjugate toward improvement of the linker structure. Bioconjug Chem 15:1264–74 15546192 10.1021/bc049868v

[CIT0044] Siegel R, Naishadham D, Jemal A. (2012). Cancer statistics, 2012. CA Cancer J Clin 62:10–29 22237781 10.3322/caac.20138

[CIT0045] Song J, Zhang Y, Zhang W, et al. (2015). Cell penetrating peptide TAT can kill cancer cells via membrane disruption after attachment of camptothecin. Peptides 63:143–9 25496911 10.1016/j.peptides.2014.12.001

[CIT0046] Song XR, Cai Z, Zheng Y, et al. (2009). Reversion of multidrug resistance by co-encapsulation of vincristine and verapamil in PLGA nanoparticles. Eur J Pharm Sci 37:300–5 19491019 10.1016/j.ejps.2009.02.018

[CIT0047] Soudy R, Chen C, Kaur K. (2013). Novel peptide–doxorubucin conjugates for targeting breast cancer cells including the multidrug resistant cells. J Med Chem 56:7564–73 24028446 10.1021/jm400647r

[CIT0048] Susa M, Iyer AK, Ryu K, et al. (2009). Doxorubicin loaded polymeric nanoparticulate delivery system to overcome drug resistance in osteosarcoma. BMC Cancer 9:399 19917123 10.1186/1471-2407-9-399 PMC2788581

[CIT0049] Szabo I, Orban E, Schlosser G, et al. (2016). Cell-penetrating conjugates of pentaglutamylated methotrexate as potential anticancer drugs against resistant tumor cells. Eur J Med Chem 115:361–8 27031212 10.1016/j.ejmech.2016.03.034

[CIT0050] Teow HM, Zhou Z, Najlah M, et al. (2013). Delivery of paclitaxel across cellular barriers using a dendrimer-based nanocarrier. Int J Pharm 441:701–11 23089576 10.1016/j.ijpharm.2012.10.024

[CIT0051] Tian XP, Qian D, He LR, et al. (2014). The telomere/telomerase binding factor PinX1 regulates paclitaxel sensitivity depending on spindle assembly checkpoint in human cervical squamous cell carcinomas. Cancer Lett 353:104–14 25045845 10.1016/j.canlet.2014.07.012

[CIT0052] Topham CH, Taylor SS. (2013). Mitosis and apoptosis: how is the balance set? Curr Opin Cell Biol 25:780–5 23890995 10.1016/j.ceb.2013.07.003

[CIT0053] Tseng Y-L, Liu J-J, Hong R-L. (2002). Translocation of liposomes into cancer cells by cell-penetrating peptides penetratin and tat: a kinetic and efficacy study. Mol Pharmacol 62:864–72 12237333 10.1124/mol.62.4.864

[CIT0054] Van Bracht E, Versteegden LR, Stolle S, et al. (2014). Enhanced cellular uptake of albumin-based lyophilisomes when functionalized with cell-penetrating peptide TAT in HeLa cells. PLoS One 9:1–9 10.1371/journal.pone.0110813 PMC4219704 25369131

[CIT0055] van Zuylen L, Nooter K, Sparreboom A, Verweij J. (2000). Development of multidrug-resistance convertors: sense or nonsense? Invest New Drugs 18:205–20 10958589 10.1023/a:1006487003814

[CIT0056] Vargas JR, Stanzl EG, Teng NN, Wender PA. (2014). Cell-penetrating, guanidinium-rich molecular transporters for overcoming efflux-mediated multidrug resistance. Mol Pharm 11:2553–65 24798708 10.1021/mp500161z PMC4123947

[CIT0057] Vasile E, Tibaldi C, Leon GL, et al. (2015). Cytochrome P450 1B1 (CYP1B1) polymorphisms are associated with clinical outcome of docetaxel in non-small cell lung cancer (NSCLC) patients. J Cancer Res Clin Oncol 141:1189–94 25504507 10.1007/s00432-014-1880-3 PMC11824122

[CIT0058] Wang C, Ma Y, Feng S, et al. (2015a). Gonadotropin-releasing hormone receptor-targeted paclitaxel-degarelix conjugate: synthesis and in vitro evaluation. J Pept Sci 21:569–76 25851250 10.1002/psc.2769

[CIT0059] Wang F, Wang Y-C, Dou S, et al. (2011). Doxorubicin-tethered responsive gold nanoparticles facilitate intracellular drug delivery for overcoming multidrug resistance in cancer cells. ACS Nano 5:3679–92 21462992 10.1021/nn200007z

[CIT0060] Wang S, Qiu J, Shi Z, et al. (2015b). Nanoscale drug delivery for taxanes based on the mechanism of multidrug resistance of cancer. Biotechnol Adv 33:224–41 25447422 10.1016/j.biotechadv.2014.10.011

[CIT0061] Wei B, Wei Y, Zhang K, et al. (2009). Development of an antisense RNA delivery system using conjugates of the MS2 bacteriophage capsids and HIV-1 TAT cell-penetrating peptide. Biomed Pharmacother 63:313–18 18823738 10.1016/j.biopha.2008.07.086

[CIT0062] Xia H, Gao X, Gu G, et al. (2011). Low molecular weight protamine-functionalized nanoparticles for drug delivery to the brain after intranasal administration. Biomaterials 32:9888–98 21937105 10.1016/j.biomaterials.2011.09.004

[CIT0063] Xia H, Gu G, Hu Q, et al. (2013). Activatable cell penetrating peptide-conjugated nanoparticles with enhanced permeability for site-specific targeting delivery of anticancer drug. Bioconjug Chem 24:419–30 23350619 10.1021/bc300520t

[CIT0064] Xu Y, Asghar S, Li H, et al. (2016). Preparation of a paclitaxel-loaded cationic nanoemulsome and its biodistribution via direct intratumoral injection. Colloids Surf B Biointerfaces 142:81–8 26938323 10.1016/j.colsurfb.2016.02.046

[CIT0065] Yusuf RZ, Duan Z, Lamendola DE. (2003). Paclitaxel resistance: molecular mechanisms and pharmacologic manipulation. Curr Cancer Drug Targets 3:1–19 12570657 10.2174/1568009033333754

[CIT0066] Zhang P, Cheetham AG, Lock LL, Cui H. (2013). Cellular uptake and cytotoxicity of drug-peptide conjugates regulated by conjugation site. Bioconjug Chem 24:604–13 23514455 10.1021/bc300585h PMC3651882

[CIT0067] Zhong J, Li L, Zhu X, et al. (2015). A smart polymeric platform for multistage nucleus-targeted anticancer drug delivery. Biomaterials 65:43–55 26142775 10.1016/j.biomaterials.2015.06.042

[CIT0068] Zhou W, Xu J, Gelston E, et al. (2015). Inhibition of Bcl-xL overcomes polyploidy resistance and leads to apoptotic cell death in acute myeloid leukemia cells. Oncotarget 6:21557–71 26188358 10.18632/oncotarget.4306 PMC4673286

[CIT0069] Zunino F, Capranico G. (1990). DNA topoisomerase II as the primary target of anti-tumor anthracyclines. Anti-Cancer Drug Des 5:307–17 1963303

[CIT0070] Zuo KQ, Zhang XP, Zou J, et al. (2010). Establishment of a paclitaxel resistant human breast cancer cell strain (MCF-7/Taxol) and intracellular paclitaxel binding protein analysis. J Int Med Res 38:1428–35 20926015 10.1177/147323001003800424

