# Supplementary material for: Cell-penetrating peptide conjugates to enhance the antitumor effect of paclitaxel on drug-resistant lung cancer
Source: Drug Deliv. 2017 May 4;24(1):752–64. doi: 10.1080/10717544.2017.1321060 (PMC8253140; doi:10.1080/10717544.2017.1321060)
Supplement: Supplemental_material.docx [file IDRD_A_1321060_SM9396.docx]

**Supplemental material**

**Cell-penetrating peptide conjugates to enhance the antitumor effect of paclitaxel on drug-resistant lung cancer**

Ziqing Duan^a^, Cuitian Chen^a^, Jing Qin^a^, Qi Liu^a^, Qi Wang^b,^ Xinchun Xu^c^*, Jianxin Wang^a^*

*^a^ Department of Pharmaceutics, School of Pharmacy, Fudan University & Key Laboratory of Smart Drug Delivery, Ministry of Education, Shanghai 201203, PR China. ^b^ Institute of Clinical Pharmacology, Guangzhou University of Traditional Chinese Medicine, Guangzhou 510006, PR China. ^c^ Shanghai Xuhui Central Hospital, Shanghai 200031, PR China*

Corresponding Authors

* Corresponding author. Xinchun Xu, Tel.: +86-21-31270810, E-mail: xuxinchun@live.cn

* Corresponding author. Jianxin Wang, Tel.: +86-21-51980088, E-mail: [jxwang@fudan.edu.cn](mailto:jxwang@fudan.edu.cn)

1. Characterization of PTX-SA using ^1^H-NMR and Q-TOF


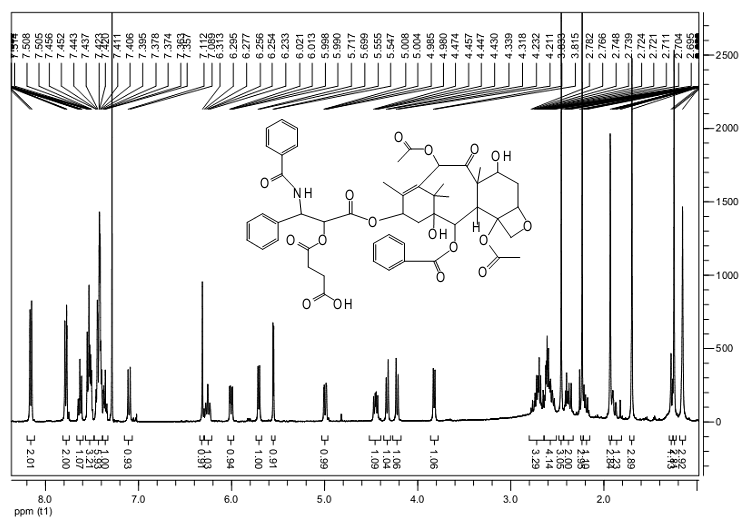


**Figure S1**. ^1^H-NMR spectrum of PTX-SA in CDCl_3_


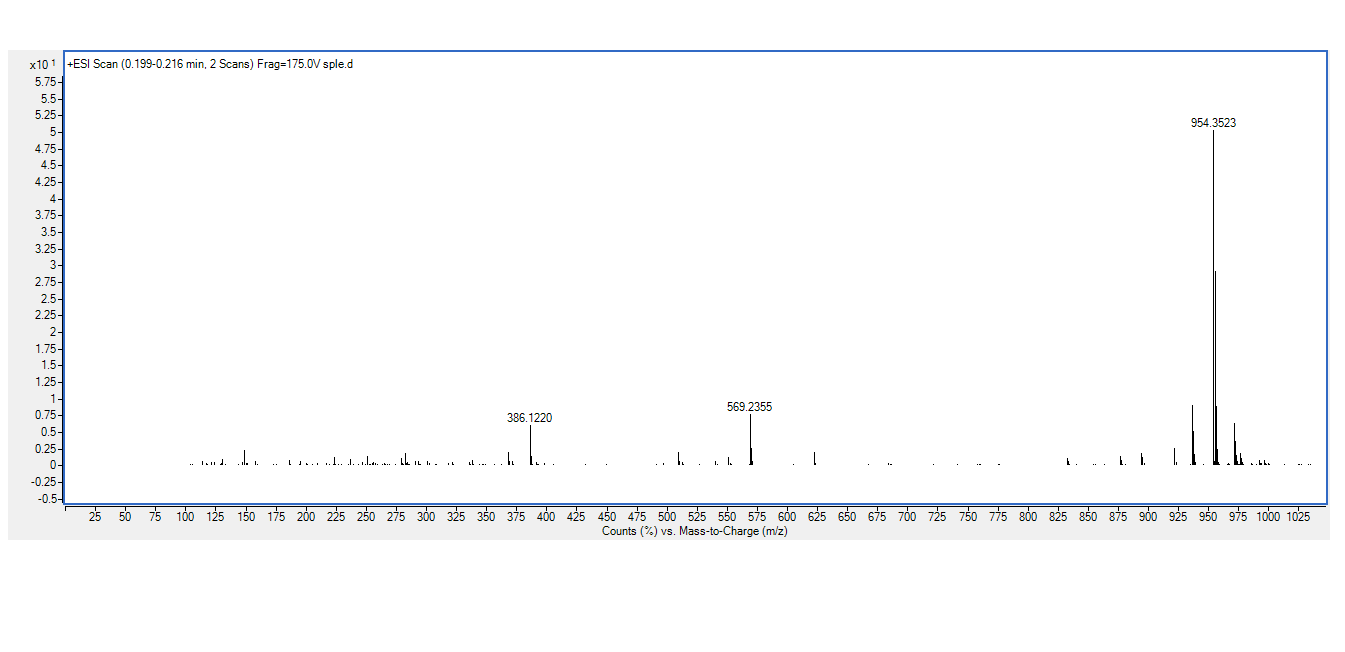


**Figure S2**. Q-TOF mass spectrometry of PTX-SA

1. Characterization of PTX-LMWP using ^1^H-NMR, MALDI-TOF and UPLC


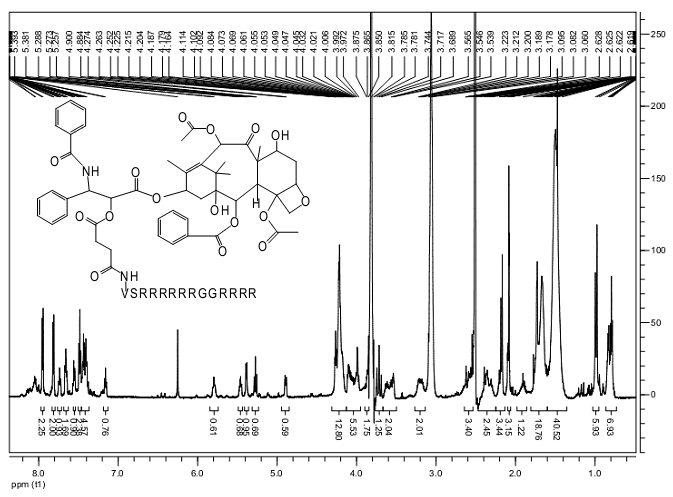


**Figure S3**. ^1^H-NMR spectrum of PTX-LMWP in D_2_O and DMSO-d6


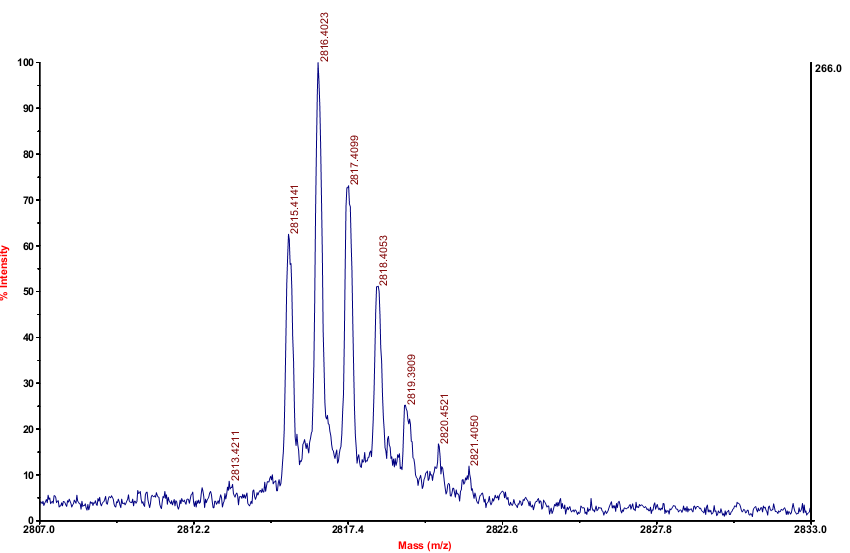


**Figure S4**. MALDI-TOF mass spectrometry of PTX-LMWP


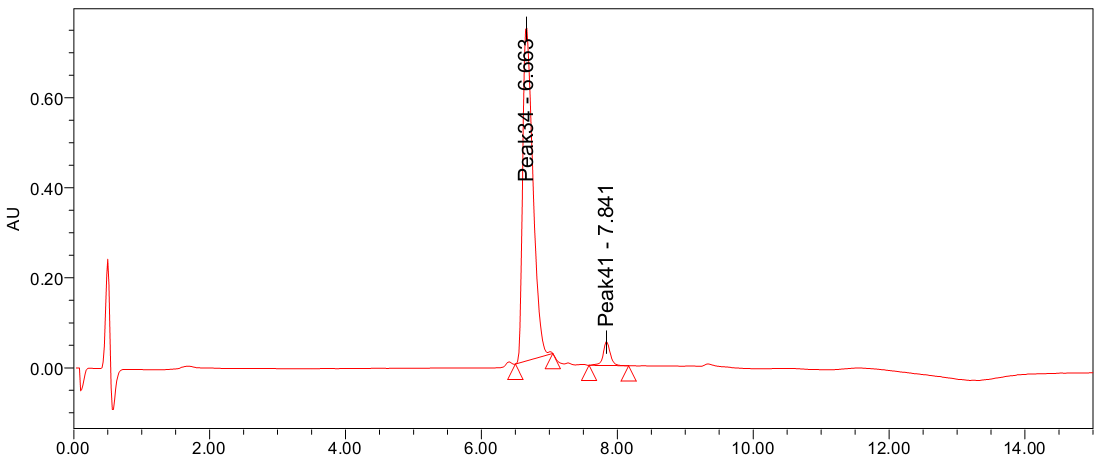


Retention time (min)

**Figure S5**. UPLC profile of PTX-LMWP

1. Characterization of PTX-SA-MAL using ^1^H-NMR and Q-TOF


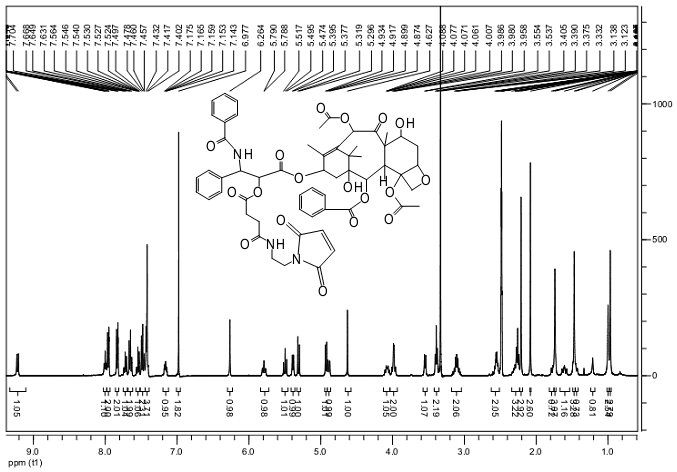


**Figure S6**. ^1^H-NMR spectrum of PTX-SA-MAL in DMSO-d6


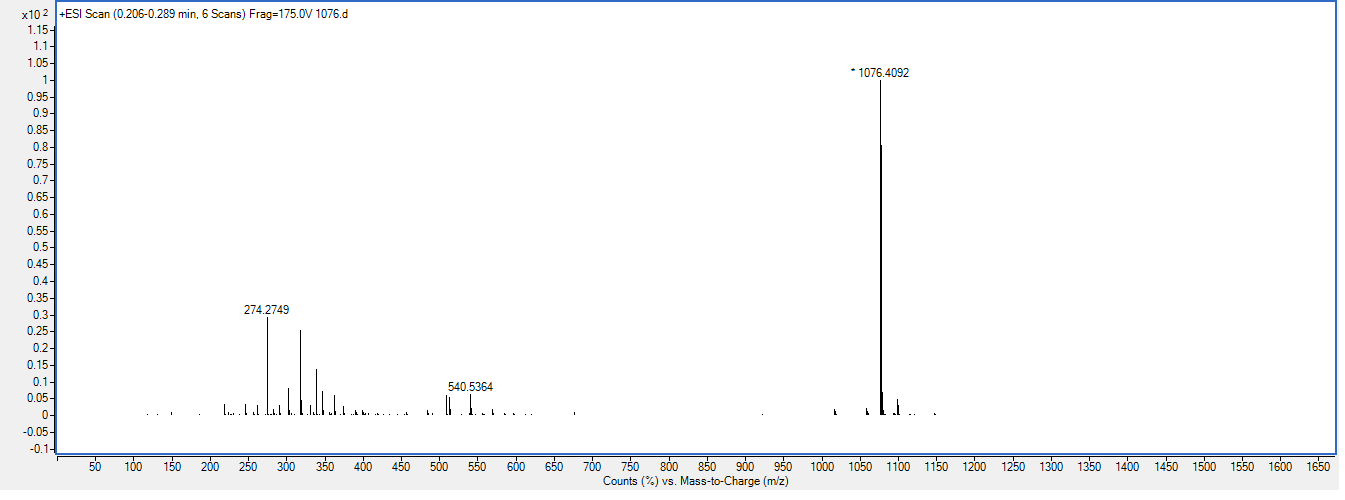


**Figure S7**. Q-TOF mass spectrometry of PTX-SA-MAL

1. Characterization of PTX-LMWP using ^1^H-NMR, MALDI-TOF and UPLC


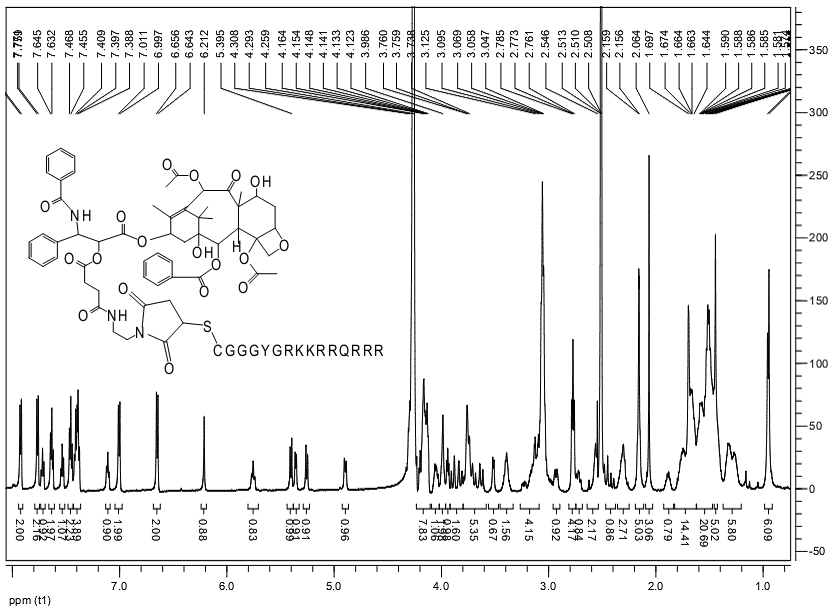


**Figure S8**. ^1^H-NMR spectrum of PTX-TAT in D_2_O and DMSO-d6


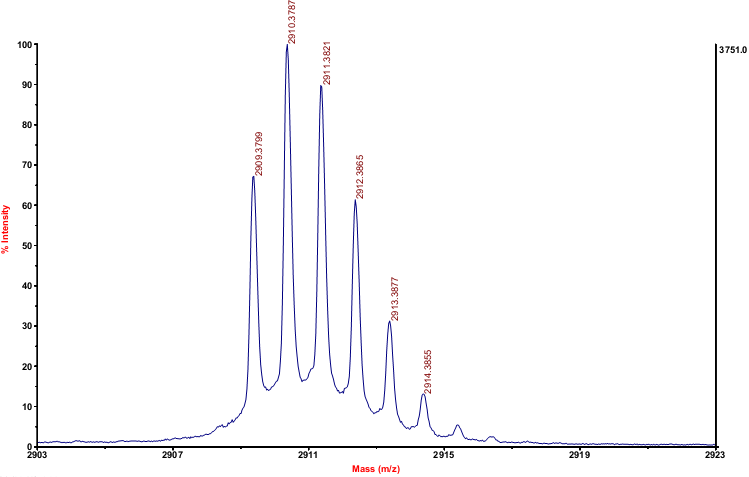


**Figure S9**. MALDI-TOF mass spectrometry of PTX-TAT


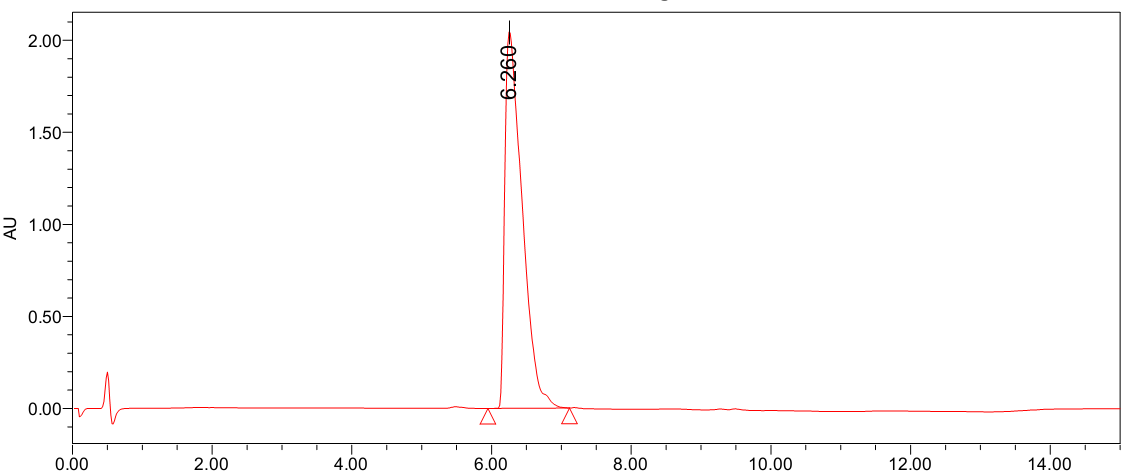


Retention time (min)

**Figure S10**. UPLC profile of PTX-TAT
